# Anthracycline’s Effects on Heart Rate Variability in Children with Acute Lymphoblastic Leukemia: Early Toxicity Signs—Pilot Study

**DOI:** 10.3390/jcm12227052

**Published:** 2023-11-12

**Authors:** Diana R. Lazar, Simona Cainap, Dana Maniu, Cristina Blag, Madalina Bota, Florin-Leontin Lazar, Alexandru Achim, Marius C. Colceriu, Mihnea Zdrenghea

**Affiliations:** 1 Department No. 11, Oncology, “Iuliu Hatieganu” University of Medicine and Pharmacy, 400012 Cluj-Napoca, Romania; 2 Department of Pediatric Cardiology, Emergency Hospital for Children, 400394 Cluj-Napoca, Romania; 3 Department of Mother and Child, “Iuliu Hatieganu” University of Medicine and Pharmacy, 400012 Cluj-Napoca, Romania; 4 Biomolecular Physics Department, Faculty of Physics, “Babes-Bolyai” University, 400347 Cluj-Napoca, Romania; 5 Department of Pediatric Oncology and Hematology, Emergency Hospital for Children, 400394 Cluj-Napoca, Romania; 6 Department No. 5, Internal Medicine, Medical Clinic Number 1, “Iuliu Hatieganu” University of Medicine and Pharmacy, 400012 Cluj-Napoca, Romania; 7 Department of Cardiology, “Niculae Stancioiu” Heart Institute, Motilor 19-21, 400001 Cluj-Napoca, Romania; 8 Department of Functional Biosciences, Discipline of Physiology, “Iuliu Hatieganu” University of Medicine and Pharmacy, 400012 Cluj-Napoca, Romania; 9 Department of Hematology, “Ion Chiricuta” Oncology Institute, 400015 Cluj-Napoca, Romania

**Keywords:** cardiotoxicity, heart rate variability, childhood cancer, early cardiac dysfunction

## Abstract

Anthracycline treatments are known to cause cardiotoxic long-term side effects in cancer survivors. Recently, a decrease in heart rate variability (HRV) has been identified in these patients, signaling autonomic dysfunction and altered cardiac fitness. This study aimed at evaluating changes in HRV in children treated with anthracyclines. A total of 35 pediatric patients with acute lymphoblastic leukemia were evaluated by means of a 24 h Holter ECG, at baseline and after reaching half the total cumulative dose of doxorubicin equivalent (120 mg/m^2^). Parameters of HRV were assessed, as well as any arrhythmic episodes, bradycardia and tachycardia percentages. The results showed a significant decrease in both time-domain and frequency-domain HRV parameters, following anthracycline treatment. The low-frequency (LF) to high-frequency (HF) parameters’ ratio also displayed a significant difference (*p* = 0.035), suggestive of early cardiac autonomic dysfunction. Of note, none of the patients presented symptoms of heart disease or elevated troponins, and only two patients presented echocardiographic signs of diastolic dysfunction. The present study showed that cardiac autonomic nervous system regulation is compromised in children treated with anthracyclines even before reaching the total cumulative dose. Therefore, HRV parameters could be the first indicators of subclinical cardiac toxicity, making Holter ECG monitoring of the oncological patient a necessity.

## 1. Introduction

Malignancies are a leading cause of pediatric disease-related deaths worldwide, with acute lymphoblastic leukemia (ALL) being one of the most frequent diagnoses (up to 80% of acute leukemias [1]) encountered in this age group. Due to the continuous research in the field, pediatric ALL cure rates have recently surpassed 90% [2], with a similar progress in event-free and overall 5-year survival rates (of more than 80%). Current treatment strategies include a very thorough patient risk stratification and treatment accordingly, with anthracyclines being one of the main treatment pillars [3]. 

Unfortunately, anthracyclines, particularly doxorubicin, have become well known for their cardiotoxic side effects, which are proportional to the total cumulative dose and treatment-free interval [4,5,6]. More than half the survivors of childhood ALL develop significant comorbidities following their oncologic treatment, with cardiac disease being one of the main long-term side effects observed [7]. Up until now, studies have focused mostly on identifying reliable cardiac biomarkers or echocardiographic parameters (7) to facilitate the detection of subclinical cardiac damage in the oncologic patient. Thus, it is known that cardiac troponins I and T and NT-proBNP are essential biomarkers to be determined during the treatment and follow-up of an oncologic patient [8,9]. Serial echocardiographic evaluations of the systolic and diastolic functions are recommended for patients undergoing oncological treatments. Recently, tissue Doppler imaging (TDI) and global longitudinal strain (GLS) have emerged as great tools in detecting subclinical diastolic disfunction induced by chemotherapeutic agents [10,11]. Also, ECG monitoring is constantly needed, as arrhythmias and even acute life-threatening events have been reported to occur even from the beginning of the oncologic treatment [12,13]. What is more, recent studies have shown that cardiotoxicity can also present itself as an alteration in cardiac autonomic function, even shortly after treatment completion [14,15].

In recent years, heart rate variability (HRV), evaluated by means of Holter ECG, has proven to be an important indicator of autonomic cardiac activity, thus revealing subtle subclinical cardiac function changes [16]. Of note, this alteration in HRV has been detected in asymptomatic patients before any changes in echocardiographic parameters [16,17]. As a consequence, HRV parameters have been proposed as a useful tool for identifying cardiac patients at risk of further developing lethal arrythmias [18,19,20]. What is more, a reduction in HRV has been described in various pathologic conditions such as diabetes [21,22], postmyocardial infarction [19,23,24,25], after cardiac transplantation [26,27], and various other neurologic [28] or endocrinologic [29] conditions, being considered a negative prognostic marker.

We further present the main findings from a series of pivotal studies on the wide application spectrum of HRV as a useful tool in the pediatric population. To begin with, cardiac autonomic dysfunction has been identified in children with cerebral palsy, with patients presenting a higher resting heart rate (HR) and a reduced HRV as compared to healthy children. Recently, Letzkus et al. have created a prediction model for cerebral palsy risk in premature infants, using HRV parameters from the early neonatal period. Their model, which exhibited an 85.3% predictability for the development of cerebral palsy, combined clinical features with HRV parameters (obtained at 1 week postpartum and 37 gestational weeks) [30]. HRV has also been intensely studied in adult patients with psychiatric disorders. Latest studies have also shown a decrease in the majority of HRV parameters in pediatric patients suffering from major depressive disorders. What is more, it was proven that depression-induced autonomic dysfunction was more detectable and substantial in children as compared to adult patients [31]. New studies even suggest the use of HRV parameters as biomarkers for depression in children/adolescents [32]. Moreover, altered HRV profiles have been detected in children suffering from autism spectrum disorders [33], as well as attention-deficit/hyperactivity disorder [34]. HRV has been evaluated in obese children, to estimate the benefit of chronic exercise exposure in these patients. A meta-analysis by Dias et al. showed that persistent exposure to exercise increased HRV in children affected by obesity, therefore enhancing the parasympathetic activity and sympatho-vagal balance [35]. What is more, in children with asthma, decreased HRV has been noticed, without correlation to disease severity or disease control [36,37]. As for pediatric cardiac disease, it has been proposed that HRV parameters could predict risk for ventricular arrhythmia development in children with viral myocarditis [38]. Last but not least, a decreased HRV has been associated with a poor outcome in children with acute heart failure [39].

Up to date, there are only a few studies to evaluate anthracycline-induced cardiotoxicity by means of 24 h Holter ECG, and current data are even more scarce in the pediatric population. It has previously been shown that doxorubicin has a negative impact on the cardiac autonomic nervous system, thus confirming its pro-arrhythmogenic and cardiotoxic effects [16]. Therefore, in this study, we aim to identify important indicators of anthracycline-induced cardiac toxicity by means of a 24 h Holter ECG in children with ALL treated with anthracycline-including protocols.

## 2. Materials and Methods

We present a single-center (Pediatric Clinic number 2, Emergency Children’s Hospital, Cluj-Napoca, Romania), investigator-driven, prospective observational study, aiming to document the pro-arrhythmogenic effects and cardiac autonomic nervous system (ANS) functioning, following anthracycline treatment in children with ALL. The objective of this study was to record the immediate and intermediate effects of anthracycline administration on heart rate variability. We obtained written informed consent from each patient and parent/legal guardian. Our study protocol was conducted in accordance with the Declaration of Helsinki and was approved by the University of Medicine and the Pharmacy “Iuliu Hatieganu” Clinical Research Ethics Committee.

In accordance with our study protocol, 35 consecutive patients were included in this study. The eligible participants were less than 18 years old at diagnosis, with a positive diagnosis of acute lymphoblastic leukemia and no history of prior heart disease. All patients were treated according to the ALL IC-BFM 2009 protocol [40], which includes anthracycline, cyclophosphamide, and methotrexate administration. Enrollment started in July 2019 and ended in July 2023, with follow-up of patients still ongoing. 

Patients who presented with a history of chest radiotherapy, a left ventricular ejection fraction (LVEF) under 50% before starting chemotherapy, or a history of other potentially cardiotoxic treatments were excluded from our study. Every patient had a complete baseline evaluation, including bedside ECG, echocardiography, and bloodwork (complete blood count, high-sensitivity C-reactive protein—hsCRP, lactate dehydrogenase—LDH), as well as the cardiac biomarkers NT-proBNP and high-sensitivity troponin (hsTroponin). 

### 2.1. Holter ECG

All children included in this study were evaluated with a bedside ECG before starting chemotherapy. Further on, to study the cardiac ANS, we performed serial Holter ECG monitoring during chemotherapy administration as follows: at the beginning of chemotherapy (the 8th day of the ALL IC-BFM 2009 protocol, before the first dose of doxorubicin equivalent) and after reaching a cumulative dose of over 120 mg/m^2^ of doxorubicin equivalent, which represents half of the total dose received per patient (day 33 of the ALL IC-BFM 2009 protocol, the end of the first anthracycline-including protocol). It is to be mentioned that the exact timeframe in between evaluations varied from patient to patient, as each had treatment delays throughout their chemotherapy protocol. What is more, the children will be re-evaluated for mid-term pro-arrhythmogenic effects during their follow-up visit on the completion of 1 year of treatment. 

The 24 h, 12-lead Holter ECG recordings were obtained using a BTL HOLTER. We extracted and analyzed the signal using the BTL Cardiopoint Holter H600 software. To begin with, all recordings were manually cleared of artifacts; then, all ectopic beats and arrhythmias were determined. The following data were obtained and analyzed: heart rate (HR), PR and QTc (corrected using the Bazett formula [41]) intervals, the percentage of tachycardia and/or bradycardia episodes, and also the presence and percentage of ventricular and supraventricular extrasystoles. 

In regard to the parameters of HRV, we evaluated frequency-domain parameters such as low-frequency (LF) and high-frequency (HF) ranges, which have been shown to corelate with cardiac ANS functioning [18]. In addition, the LF/HF ratio was calculated. The time-domain parameters evaluated included the standard deviation of all normal sinus RR intervals over 24 h (SDNN), the standard deviation of the average normal sinus RR intervals for all 5 min segments (SDANN), the root mean square of the successive normal sinus RR interval difference (rMSSD), and the percentage of successive normal sinus RR intervals over 50 milliseconds (pNN50). 

This study’s primary endpoint was cardiac ANS dysfunction, assessed through HRV parameters. The secondary endpoints were represented by the detection of arrhythmic episodes, bradycardia and tachycardia percentages, heart rate, PR interval, and QTc interval. 

### 2.2. Statistical Analysis

The main characteristics of the population under study were analyzed using Microsoft Excel 2019; then, a chi-square test for associations was performed. The clinical characteristics and heart rate variability were reported as mean ± standard deviation (sd). The two-sample paired t-test was used to determine the *p*-values corresponding to treatment-related changes for the clinical features and heart rate variability. The Pearson correlation coefficients (r) and accompanying *p*-values were determined. The calculated *p*-values were two-sided, and a *p*-value less than 0.05 was considered statistically significant. 

## 3. Results 

The present study enrolled 35 consecutive children diagnosed with ALL (mean age at diagnosis 5.97 ± 3.76 years), most of them belonging to the 3–6-year-old age group. There were slightly more boys than girls, with an almost equal urban–rural distribution. All patient characteristics are presented in Table 1.

Three patients died from sepsis and respiratory failure during our study, and two patients continued their treatment abroad. Of note, the selected patients had no pre-existing disease, nor had they followed any chronic treatment before starting chemotherapy. 

The main baseline biologic, echocardiographic, and ECG parameters are summarized in Table 2. 

Regarding the baseline biologic evaluation, most of the patients presented with leukocytosis (75.76%), anemia (87.87%), and thrombocytopenia (90.9%). It is to be noted that some presented with values higher than 50,000 leukocytes/mm^3^ (21.21%), some with severe anemia with hemoglobin values lower than 7 g/dL (39.39%), and a lot of patients with severe thrombocytopenia with thrombocyte values under 30,000/mm^3^ (45.45%). A big percentage of our patients (42.42%) presented with hsCRP within the normal range (<1 mg/dL), but amongst the ones with increased hsCRP, the majority (58%) presented values above 3 mg/dL. 

**Figure 1:**
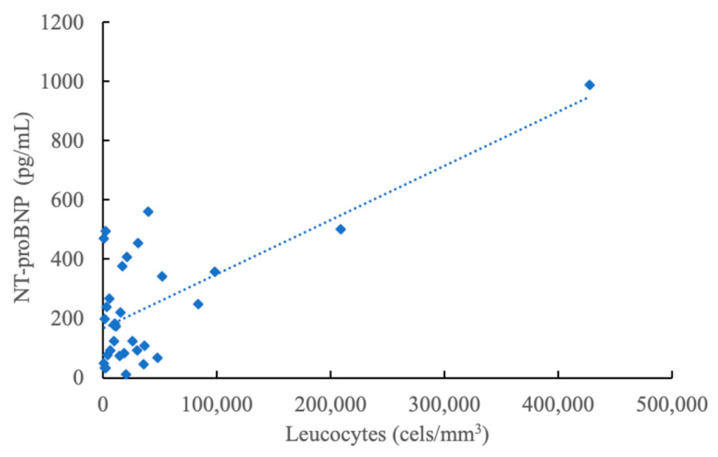
Correlation between NT-proBNP and leucocyte values at diagnosis.

The baseline evaluation also revealed that a big percentage of our patients (40.63%) exhibited a reacted NT-proBNP before the onset of chemotherapy, whereas none presented elevated troponin values at diagnosis. The baseline NT-proBNP values showed a moderate positively correlation (Figure 1) with the baseline leukocyte values (r Pearson coefficient 0.66, *p* < 0.001). Evaluation of the cardiac biomarkers after reaching half the total cumulative dose of anthracycline revealed a statistically significant increase in hsTroponin values (mean ± standard deviation = 9.23 ± 9.62; *p*-value = 0.007). However, the obtained values do not surpass our laboratory cutoff limit (14 ng/L); therefore, they cannot be interpreted as pathological. Also, the NT-proBNP values did not change significantly at the second evaluation (mean ± standard deviation = 629.81 ± 956.99; *p*-value = 0.06).

The baseline bedside ECG evaluation detected 11.54% of patients with resting HR above the 98th percentile for age and sex, with only 3.85% of patients presenting with values below the second percentile for age and sex. Moreover, none of our patients had an increased PR interval at diagnosis, but almost 35% of them had QTc interval values outside the reference range for age and sex.

As for the initial echocardiographic evaluation, an LVEF under 60% (but over 50%) at diagnosis was described in only two patients. Regarding the TDI evaluation of diastolic function, 69.57% of patients presented E/A values within the 10th–90th percentile for age, while only 17.39% of patients presented E/E′ values corresponding to the 10th–90th percentile interval for age. However, E/A values indicative of a restrictive LV filling pattern and a pseudo-normal LV filling pattern were reported for only two and one patient, respectively. Regarding the E/E′ ratio, there were no patients with E/E′ values higher than the adult cutoff limit suggestive of increased LV filling pressures. The second echocardiographic evaluation performed after reaching 120 mg/m^2^ of doxorubicin equivalent did not reveal statistically or clinically significant changes from the baseline. There were no significant alterations in diastolic or systolic functions, no kinetic modifications or volume changes.

The 24 h ambulatory ECG monitoring was performed at the beginning of chemotherapy(baseline) and after half of the total cumulative dose of doxorubicin equivalent (120 mg/m^2^), with the main findings being summarized in Table 3.

When comparing the two datasets, a statistically significant increase in heart rate (*p* < 0.001), as well a statistically significant reduction in bradycardia percentage (*p* = 0.011), was observed. Of note, the PR interval significantly increased (*p* = 0.012); however, no AV blocks, sinus pauses, or significant arrythmias were identified. Also, the QTc interval values did not vary significantly amongst examinations.

Regarding the HRV parameters, statistically significant changes were detected both in the time-domain and the frequency-domain evaluated parameters. The results indicated a meaningful (*p* < 0.05) reduction in all the time-domain variables (SDANN, SDANN, rMSSD, and pNN50), after reaching 120 mg/m^2^ of doxorubicin equivalent, as compared to the baseline. The same was noted for the frequency-domain characteristics determined (LF, HF), with similar statistically significantly (*p* < 0.05) lower values after reaching half the total cumulative dose as compared to the baseline evaluation. The LF/HF ratio showed a statistically significant increase in values following chemotherapy administration.

There were no clinically significant arrhythmia episodes, nor did the incidence of supraventricular or ventricular extrasystoles vary statistically significant amongst examinations.

## 4. Discussion

This study investigated the effects of anthracycline treatments on cardiac function, particularly the cardiac autonomic system, by means of 24 h Holter ECG evaluations. It is to be noted that there are currently only limited data derived from few studies regarding the HRV effects of anthracycline treatment in children [16,22]. 

Regarding the baseline evaluation of our patients, we validated (r = 0.66, *p* < 0.001) the results obtained in a previous study [42], in which we identified a positive linear correlation between leukocyte values at diagnosis and NT-proBNP values. Therefore, we once again indulged the hypothesis that even before treatment, cardiac function is slightly influenced by either direct infiltration of malignant clones in the cardiac tissue or by the overall pro-inflammatory state of the oncologic patient. HsCRP values are universally used to evaluate patients’ inflammatory status. What is more, a value of over 3 mg/dL has been shown to correlate with an increased risk of acute cardiac events, particularly sudden cardiac death [43]. Therefore, even though oncologic patients are known to exhibit a pro-inflammatory status, it is important to periodically monitor their hsCRP values and to correlate these values with the cardiac function.

Doxorubicin has been shown to increase sympathetic activity shortly after administration, thus correlating with the increased acute pro-arrhythmogenic risk identified in some studies [12]. Life-threatening arrhythmias are, however, extremely rare, with most studies reporting an increase in supraventricular tachycardia incidence and a prolonged QT interval following treatment [13,44]. In our study, the number of supraventricular or ventricular extrasystoles occurring after the first anthracycline–including protocol (corresponding to half the total cumulative dose) was within the reference range for age. What is more, there was no statistically significant QT prolongation, although a big percentage of patients had QTc values that were above the reference range for age and sex at their baseline ECG evaluation. 

However, it is to be noted that these results were obtained after only half of the total cumulative dose given per patient. Another Holter ECG evaluation is required after reaching the total cumulative dose (240 mg/m^2^—according to the ALL IC BFM 2009 protocol). As shown in previous studies, HRV deterioration continued to persist until the end of chemotherapy, even without significant echocardiographic changes. Stachowiak et al. noticed in their study a correlation between increasing NT-proBNP values throughout chemotherapy and decreasing HRV [14]. In our study, however, the NT-proBNP values did not correlate with the HRV parameters. We expect that, after chemotherapy cessation, the HRV parameters will continue to decrease, and a correlation with the NT-proBNP values will be re-evaluated at the end of the oncologic treatment.

As has been demonstrated, heart rate variability reflects the autonomic cardiac function [18], with more and more studies emphasizing the important prognostic value of HRV parameters in various cardiac pathologies. Zeid et al. demonstrated that a decrease in HRV parameters (particularly nonlinear power spectral density parameters) was the strongest, independent mortality predictor in patients with heart failure [45]. Also, in patients with myocardial infarction, depressed HRV parameters have been shown to be a powerful tool in predicting mortality and arrhythmic complications [23,24]. Recent studies on anthracycline’s effects on HRV have shown a decrease in all HRV parameters following treatment, which does not seem to improve with time from treatment cessation [14,16]. Also, in vivo studies on rodents confirmed the negative impact doxorubicin has on the cardiac ANS [46]. What is more, Caru et al. demonstrated the protective effects of dexrazoxane on cardiac ANS functioning after high-dose anthracycline treatment, thus emphasizing the importance of using cardioprotective agents in patients treated with cardiotoxic drugs [16]. 

Our study has proven that, even after half the total cumulative dose, both time-domain and frequency-domain HRV parameters were significantly lower than at baseline. This is consistent with other findings so far [14,15,16]. As other research has indicated, frequency-domain parameters are a more accurate indicator for CANS function [15,47]. It has been proven that, the LF/HF ratio is an important marker of sympathetic–parasympathetic balance [41] and has also been clearly correlated to heart failure progression [42,43]. As such, our study demonstrates a significant decrease in LF and HF with an increase in the LF/HF ratio, respectively. This unveils an early dysfunction of the cardiac ANS owing to increased sympathetic modulation. It could be hypothesized that this sympathetic dominance could be an indicator of an early-on compensatory response to abnormal cardiac functioning. Therefore, it could be stated that a decrease in HRV is an early indicator of altered cardiac function, being one of the very first noticeable signs of anthracycline-induced cardiac toxicity. 

## 5. Limitations

Our study has been limited by the small number of patients; a larger, multi-centric study being needed to establish appropriate Holter-ECG monitoring intervals for children treated with anthracyclines. Also, as compared to other studies, we did not use cardioprotective agents (such as dexrazoxane), as they are not approved for use in the pediatric population in our country. What is more, these results were obtained mid-treatment after only half of the total cumulative doxorubicin equivalent dose had been given. A follow-up of these patients, with Holter ECG evaluation after reaching the total cumulative dose and 1 year after treatment cessation is to be performed.

## 6. Conclusions

In our study, pediatric patients diagnosed with ALL displayed a significant decrease in HRV parameters induced by anthracycline treatment. Our findings are consistent with the existing literature, according to which the regulation of cardiac ANS is compromised in children treated with anthracyclines even before reaching the total cumulative dose. These findings emphasize the importance of cardiac monitoring during the oncological treatment, as Holter ECG monitoring could be used to determine early cardiotoxicity signs and the need for treatment. Further studies are needed to establish the exact implications of these results for the long-term cardiac function and cardiotoxicity prophylaxis.

## Figures and Tables

**Table 1:** Patient characteristics.

	Mean ± SD	n (%)
Age at diagnosis (years)	5.97 ± 3.76	
1–3 years		8 (22.86%)
3–6 years		13 (37.14%)
6–10 years		9 (25.71%)
10–18 years		5 (14.29%)
Gender		
Boys		18 (51.43%)
Girls		17 (48.57%)
Weight (kg)	23.02 ± 13.46	
Height (m)	115.5 ± 26.09	
Body mass index (kg/m^2^)	15.84 ± 2.18	
underweight		12 (34.29%)
normal		19 (54.29%)
overweight		3 (8.57%)
obese		2 (2.86%)

**Table 2:** Baseline characteristics.

Parameter	Mean ± Standard Deviation
Baseline laboratory parameters	
Leukocytes (cells/mm^3^)	54,002.42 ± 93,197.84
Hemoglobin (g/dL)	7.23 ± 2.81
Thrombocytes (cells/mm^3^)	58,912.82 ± 80,779.52
LDH (U/L)	846.48 ± 1632.77
hsCRP (mg/dL)	5.13 ± 15.24
NT-proBNP (pg/mL)	597.06 ± 952.46
hsTroponin (ng/L)	3.71 ± 2.1
Baseline ECG	
HR (bpm)	95.88 ± 26.59
PR interval (ms)	122.96 ± 20.27
QTc interval (ms)	428.46 ± 39.93
Baseline echocardiography	
LVEF (%)	67.68 ± 6.64
LVSF (%)	36.33 ± 5.77
E/A	1.85 ± 0.27
E/E′	7.37 ± 2.63

**Table 3:** Changes in Holter ECG parameters after half the total cumulative dose of anthracycline.

Parameter	Baseline	After 120 mg/m^2^ of Doxorubicin Equivalent	*p*-Value
HR	87.28 ± 13.8	103.2 ± 21.8	**<0.001**
Tachycardia (%)	7.79 ± 11.96	15.44 ± 22.26	0.19
Bradycardia (%)	30.07 ± 26.57	18.77 ± 22.56	**0.011**
PR interval (ms)	121.28 ± 19.3	135.58 ± 17.14	**0.012**
QTc (ms)	458.62 ± 73.6	442 ± 27.56	0.61
Time-domain parameters			
SDNN (ms)	157.66 ± 49.37	114.95 ± 54.63	**0.002**
SDANN (ms)	125.48 ± 43.12	97.7 ± 47.61	**0.007**
rMSSD (ms)	109.76 ± 51.8	63.85 ± 42.35	**0.003**
pNN50 (%)	36.65 ± 18.87	17.84 ± 16.96	**<0.001**
Frequency-domain parameters			
LF (ms)	0.47 ± 0.15	0.34 ± 0.15	**0.019**
HF (ms)	0.8 ± 0.35	0.51 ± 0.31	**0.018**
LF/HF ratio	0.65 ± 0.2	0.82 ± 0.32	**0.036**

Data are shown as mean ± standard deviation. Bolded *p* values (p<0.05) are representative of statistical significant changes. HRV = heart rate variability; ALL = acute lymphoblastic leukemia SDNN = standard deviation of all normal sinus RR intervals over 24 h; SDANN = standard deviation of the average normal sinus RR intervals for all 5 min segments over 24 h; rMSSD = root mean square of the successive normal sinus RR interval difference; pNN50 = percentage of successive normal sinus RR intervals over 50 milliseconds; LF = low frequency; HF = high frequency.

## Data Availability

The data presented in this study are available on request from the corresponding author. The data are not publicly available because of GPDR legislation.

## References

[1] Brown P., Inaba H., Annesley C., Beck J., Colace S., Dallas M., DeSantes K., Kelly K., Kitko C., Lacayo N. (2020). Pediatric Acute Lymphoblastic Leukemia, Version 2.2020, NCCN Clinical Practice Guidelines in Oncology. J. Natl. Compr. Cancer Netw..

[2] Hocking J., Schwarer A.P., Gasiorowski R., Patil S., Avery S., Gibson J., Iland H., Ho P.J., Joshua D., Muirhead J. (2014). Excellent outcomes for adolescents and adults with acute lymphoblastic leukemia and lymphoma without allogeneic stem cell transplant: The FRALLE-93 pediatric protocol. Leuk. Lymphoma.

[3] Campbell M., Kiss C., Zimmermann M., Riccheri C., Kowalczyk J., Felice M.S., Kuzmanovic M., Kovacs G., Kosmidis H., Gonzalez A. (2023). Childhood Acute Lymphoblastic Leukemia: Results of the Randomized Acute Lymphoblastic Leukemia Intercontinental-Berlin-Frankfurt-Münster 2009 Trial. JCO.

[4] Nebigil C.G., Désaubry L. (2018). Updates in Anthracycline-Mediated Cardiotoxicity. Front. Pharmacol..

[5] Lipshultz S.E., Cochran T.R., Franco V.I., Miller T.L. (2013). Treatment-related cardiotoxicity in survivors of childhood cancer. Nat. Rev. Clin. Oncol..

[6] Tilemann L.M., Heckmann M.B., Katus H.A., Lehmann L.H., Müller O.J. (2018). Cardio-oncology: Conflicting priorities of anticancer treatment and cardiovascular outcome. Clin. Res. Cardiol..

[7] Lipshultz S.E., Colan S.D., Gelber R.D., Perez-Atayde A.R., Sallan S.E., Sanders S.P. (1991). Late Cardiac Effects of Doxorubicin Therapy for Acute Lymphoblastic Leukemia in Childhood. N. Engl. J. Med..

[8] Ananthan K., Lyon A.R. (2020). The Role of Biomarkers in Cardio-Oncology. J. Cardiovasc. Trans. Res..

[9] Joolharzadeh P., Rodriguez M., Zaghlol R., Pedersen L.N., Jimenez J., Bergom C., Mitchell J.D. (2023). Recent Advances in Serum Biomarkers for Risk Stratification and Patient Management in Cardio-Oncology. Curr. Cardiol. Rep..

[10] Cannizzaro M.T., Inserra M.C., Passaniti G., Celona A., D’Angelo T., Romeo P., Basile A. (2023). Role of advanced cardiovascular imaging in chemotherapy-induced cardiotoxicity. Heliyon.

[11] Makavos G., Ikonomidis I., Palios J., Rigopoulos A., Katogiannis K., Parissis J., Paraskevaidis I., Noutsias M. (2021). Cardiac imaging in cardiotoxicity: A focus on clinical practice. Heart Fail. Rev..

[12] Benjanuwattra J., Siri-Angkul N., Chattipakorn S.C., Chattipakorn N. (2020). Doxorubicin and its proarrhythmic effects: A comprehensive review of the evidence from experimental and clinical studies. Pharmacol. Res..

[13] Li H., Yang W., Yu Z., Peng Y., Huang M., Long L., Lu A., Tan L., Deng M., Qu H. (2022). Risk of Arrhythmia with Exposure to Anthracyclines: A Systematic Review, Meta-Analysis, and Network Meta-Analysis. In Review. https://www.researchsquare.com/article/rs-2318045/v1.

[14] Stachowiak P., Milchert-Leszczyńska M., Falco M., Wojtarowicz A., Kaliszczak R., Safranow K., Kornacewicz-Jach Z. (2018). Heart rate variability during and after chemotherapy with anthracycline in patients with breast cancer. Kardiol. Pol..

[15] Nevruz O., Yokusoglu M., Uzun M., Demirkol S., Avcu F., Baysan O., Koz C., Cetin T., Sag C., Ural A.U. (2007). Cardiac Autonomic Functions are Altered in Patients with Acute Leukemia, Assessed by Heart Rate Variability. Tohoku J. Exp. Med..

[16] Caru M., Corbin D., Périé D., Lemay V., Delfrate J., Drouin S., Bertout L., Krajinovic M., Laverdière C., Andelfinger G. (2019). Doxorubicin treatments induce significant changes on the cardiac autonomic nervous system in childhood acute lymphoblastic leukemia long-term survivors. Clin. Res. Cardiol..

[17] Viniegra M., Marchetti M., Losso M., Navigante A., Litovska S., Senderowicz A., Borghi L., Lebron J., Pujato D., Marrero H. (1990). Cardiovascular autonomic function in anthracycline-treated breast cancer patients. Cancer Chemother. Pharmacol..

[18] Malik M., Bigger J.T., Camm A.J., Kleiger R.E., Malliani A., Moss A.J., Schwartz P.J. (1996). Heart rate variability: Standards of measurement, physiological interpretation, and clinical use. Eur. Heart J..

[19] Faber T.S., Staunton A., Hnatkova K., Camm A.J., Malik M. (1996). Stepwise strategy of using short- and long-term heart rate variability for risk stratification after myocardial infarction. Pacing Clin. Electrophysiol..

[20] Huikuri H.V., Stein P.K. (2013). Heart rate variability in risk stratification of cardiac patients. Prog. Cardiovasc. Dis..

[21] Benichou T., Pereira B., Mermillod M., Tauveron I., Pfabigan D., Maqdasy S., Dutheil F. (2018). Heart rate variability in type 2 diabetes mellitus: A systematic review and meta–analysis. PLoS ONE.

[22] Christoffersen L., Gibson T.M., Pui C., Joshi V., Partin R.E., Green D.M., Lanctot J.Q., Howell C.R., Mulrooney D.A., Armstrong G.T. (2020). Cardiac autonomic dysfunction in survivors of childhood acute lymphoblastic leukemia: The St. Jude Lifetime Cohort Study. Pediatr. Blood Cancer.

[23] Buccelletti E., Gilardi E., Scaini E., Galiuto L., Persiani R., Biondi A., Basile F., Silveri N.G. (2009). Heart rate variability and myocardial infarction: Systematic literature review and metanalysis. Eur. Rev. Med. Pharmacol. Sci..

[24] Stein P.K., Domitrovich P.P., Huikuri H.V., Kleiger R.E. (2005). Cast Investigators Traditional and nonlinear heart rate variability are each independently associated with mortality after myocardial infarction. J. Cardiovasc. Electrophysiol..

[25] Mäntysaari M., Kuikka J., Hartikainen J., Mustonen J., Mussalo H., Tahvanainen K., Länsimies E., Uusitupa M., Pyörälä K. (1995). Myocardial sympathetic nervous dysfunction detected with iodine-123-MIBG is associated with low heart rate variability after myocardial infarction. J. Nucl. Med..

[26] Ramaekers D., Ector H., Vanhaecke J., van Cleemput J., van de Werf F. (1996). Heart rate variability after cardiac transplantation in humans. Pacing Clin. Electrophysiol..

[27] Sanatani S., Chiu C., Nykanen D., Coles J., West L., Hamilton R. (2004). Evolution of heart rate control after transplantation: Conduction versus autonomic innervation. Pediatr. Cardiol..

[28] da Silva T.D., Massetti T., Crocetta T.B., de Mello Monteiro C.B., Carll A., Vanderlei L.C.M., Arbaugh C., Oliveira F.R., de Abreu L.C., Ferreira Filho C. (2018). Heart Rate Variability and Cardiopulmonary Dysfunction in Patients with Duchenne Muscular Dystrophy: A Systematic Review. Pediatr. Cardiol..

[29] Brusseau V., Tauveron I., Bagheri R., Ugbolue U.C., Magnon V., Bouillon-Minois J.-B., Navel V., Dutheil F. (2022). Heart Rate Variability in Hyperthyroidism: A Systematic Review and Meta-Analysis. Int. J. Environ. Res. Public Health.

[30] Letzkus L., Picavia R., Lyons G., Brandberg J., Qiu J., Kausch S., Lake D., Fairchild K. (2023). Heart rate patterns predicting cerebral palsy in preterm infants. Pediatr. Res..

[31] Chen W., Zhong Q., Chen H., Chen S. (2023). Heart rate variability in children and adolescents with major depressive disorder: A systematic review and meta-analysis. J. Affect. Disord..

[32] Baumeister-Lingens L., Rothe R., Wolff L., Gerlach A.L., Koenig J., Sigrist C. (2023). Vagally-mediated heart rate variability and depression in children and adolescents—A meta-analytic update. J. Affect. Disord..

[33] Thapa R., Pokorski I., Ambarchi Z., Thomas E., Demayo M., Boulton K., Matthews S., Patel S., Sedeli I., Hickie I.B. (2021). Heart Rate Variability in Children With Autism Spectrum Disorder and Associations With Medication and Symptom Severity. Autism Res..

[34] Bellato A., Arora I., Kochhar P., Ropar D., Hollis C., Groom M.J. (2022). Heart Rate Variability in Children and Adolescents with Autism, ADHD and Co-occurring Autism and ADHD, During Passive and Active Experimental Conditions. J. Autism Dev. Disord..

[35] Dias R.M., Moraes Í.A.P., Dantas M.T.A.P., Fernani D.C.G.L., Fontes A.M.G.G., Silveira A.C., Barnabé V., Fernandes M., Martinelli P.M., Monteiro C.B.M. (2021). Influence of Chronic Exposure to Exercise on Heart Rate Variability in Children and Adolescents Affected by Obesity: A Systematic Review and Meta-Analysis. Int. J. Environ. Res. Public Health.

[36] Franco O.S., Júnior A.O.S., Signori L.U., Prietsch S.O.M., Zhang L. (2020). Cardiac autonomic modulation assessed by heart rate variability in children with asthma. Pediatr. Pulmonol..

[37] Schiwe D., Vendrusculo F.M., Becker N.A., Donadio M.V.F. (2023). Impact of asthma on heart rate variability in children and adolescents: Systematic review and meta-analysis. Pediatr. Pulmonol..

[38] Ling N., Li C.-L., Wang Z.-Z., Zhang H.-N., Xu H., An X.-J. (2018). Heart rate variability in children with myocarditis presenting with ventricular arrhythmias. Eur. Rev. Med. Pharmacol. Sci..

[39] Connell P.S., Price J.F., Rusin C.G., Howard T.S., Spinner J.A., Valdes S.O., Pham T.D.N., Miyake C.Y., Kim J.J. (2023). Decreased Heart Rate Variability in Children with Acute Decompensated Heart Failure is Associated with Poor Outcomes. Pediatr. Cardiol..

[40] Campbell D.M. (2009). All IC-BFM 2009—Trial Steering Committee. https://www.google.com.hk/url?sa=t&rct=j&q=&esrc=s&source=web&cd=&ved=2ahUKEwiv4t2XiLuCAxV_plYBHROIAmEQFnoECBcQAQ&url=https%3A%2F%2Fwww.bialaczka.org%2Fwp-content%2Fuploads%2F2016%2F10%2FALLIC_BFM_2009.pdf&usg=AOvVaw13HbMigLHAYgMqIeqtGwhK&opi=89978449.

[41] Bazett H.C. (1997). An Analysis of the Time-Relations of Electrocardiograms. Ann. Noninvasive Electrocardiol..

[42] Maniu D.R., Blag C., Popa G., Bota M., Vlad C., Cainap C., Balacescu O., Pop L., Cainap S.S. (2017). The Role of Biomarkers and Echocardiography in the Evaluation of Cardiotoxicity Risk in Children Treated for Leukemia.

[43] Zhou R., Xu J., Luan J., Wang W., Tang X., Huang Y., Su Z., Yang L., Gu Z. (2022). Predictive role of C-reactive protein in sudden death: A meta-analysis of prospective studies. J. Int. Med. Res..

[44] Volkova M., Russell R. (2011). Anthracycline Cardiotoxicity: Prevalence, Pathogenesis and Treatment. Curr. Cardiol. Rev..

[45] Zeid S., Buch G., Velmeden D., Söhne J., Schulz A., Schuch A., Tröbs S.-O., Heidorn M.W., Müller F., Strauch K. (2023). Heart rate variability: Reference values and role for clinical profile and mortality in individuals with heart failure. Clin. Res. Cardiol..

[46] Potočnik N., Perše M., Cerar A., Injac R., Finderle Ž. (2017). Cardiac autonomic modulation induced by doxorubicin in a rodent model of colorectal cancer and the influence of fullerenol pretreatment. PLoS ONE.

[47] Kirizawa J.M., Garner D.M., Arab C., Valenti V.E. (2020). Is heart rate variability a valuable method to investigate cardiac autonomic dysfunction in subjects with leukemia? A systematic review to evaluate its importance in clinical practice. Support. Care Cancer.

